# Correlation Analysis between Mechanical Properties and Fractions Composition of Oil-Rejuvenated Asphalt

**DOI:** 10.3390/ma15051889

**Published:** 2022-03-03

**Authors:** Rongyan Tian, Haoyuan Luo, Xiaoming Huang, Yangzezhi Zheng, Leyi Zhu, Fengyang Liu

**Affiliations:** 1 School of Transportation, Southeast University, Nanjing 211189, China; 230179751@seu.edu.cn (R.T.); huangxm@seu.edu.cn (X.H.); 230208809@seu.edu.cn (Y.Z.); 220213392@seu.edu.cn (L.Z.); 220213385@seu.edu.cn (F.L.); 2 National Demonstration Center for Experimental Education of Road and Traffic Engineering, Southeast University, Nanjing 211189, China; 3 College of Engineering, Tibet University, Lhasa 850000, China

**Keywords:** asphalt binder, fractions composition, mechanical property, rheological properties, asphalt colloidal structure, asphaltene

## Abstract

To clarify the intrinsic relationship between the mechanical properties of asphalt and its fraction composition, the SARA fraction composition and six macroscopic mechanical properties (critical cracking temperature (*T_CR_*), fatigue life (*N_f_*), non-recoverable creep (*J*_*nr*3.2_), penetration, ductility, and softening point) were investigated for 16 asphalt samples. Fraction contents of asphaltene and aromatic are strongly correlated with *T_CR_* and ductility (*R*^2^ > 0.92) that characterize the ability of asphalt to adapt to deformation at low and medium temperatures. Heavy fraction (asphaltene and resins) content is also strongly correlated with (*R*^2^ > 0.90) penetration and *J*_*nr*3.2_ that characterize the resistance of the asphalt to overall deformation at medium and high temperatures. To express the changes in the four fractions simultaneously with one indicator, a statistic, *average deviation of the fractions between the given asphalt and its original* (marked *σ*), is introduced in this study to characterize the degree of asphalt aging based on the fraction changes. It normalizes the four simultaneous change indicators (percentage of SARA fractions) during asphalt aging into one indicator. This new indicator has a strong correlation with several mechanical performance indicators of asphalt, where it is strongly correlated with *T_CR_* (*R*^2^ > 0.90), ductility, and penetration, which are also well correlated with *J*_*nr*3.2_ (*R*^2^ > 0.85), *N_f_* (*R*^2^ > 0.75), and softening point (*R*^2^ > 0.75).

## 1. Introduction

The asphalt binder is the residue from petroleum refining and has been used as a modern road material for 200 years owing to its suitable viscoelastic behavior. A quality asphalt binder is expected to have sufficient toughness, adaptability to deformation at low temperatures [1], sufficient modulus, and elastic recovery at high temperatures [2]. Moreover, it should be able to quickly absorb the energy from repetitive loads and release stresses during the temperature range in which it normally operates [3]. Subtle differences in crude oil origin, refining procedures, additives, and other factors can affect these mechanical properties. The basis for the differences in the external mechanical properties of asphalt is the difference in its internal chemical composition [4]. Scholars have sought to understand the fractions of the compounds that make up asphalt binders to redesign their entire structure [5] and to regulate their external mechanical properties [6] to produce asphalt binders that can be used in various complex environments [7].

The compounds within the asphalt include various n-/isomeric alkanes, aliphatic chain compounds, cyclic aromatic compounds, thick-ringed aromatic hydrocarbons, and various heteroatoms (such as nitrogen, sulfur, and iron) [8]. Many of these compounds are complex in structure and similar in molecular mass and chemical properties, making it difficult to separate and quantify them with the existing technical procedure [9]. A common and accepted practice is to divide the asphalt into four fractions according to molecular weight (SARA), namely Saturates (S), Aromatics (A), Resins (R), and Asphaltenes (As) [10]. Saturates have the lowest molecular masses among the four, their average molar mass ranges from approximately 470 to 800 g/mol, and their main components are waxy aliphatic chain compounds containing small amounts of linear n-alkanes and few aromatic rings [11]. Aromatic fractions are mainly composed of cyclic aromatic compounds with a molar mass between 600 and 980 g/mol [8]. Resins consist mainly of aromatic compounds with polar hybridized sulfur and nitrogen atoms, and their chemical composition is very close to that of asphaltenes, with only slightly lower molecular weights and polarities [12]. Asphaltenes have the highest polarity, with many thick-ringed aromatic compounds and various types of heteroatoms, and their molar mass usually ranges from approximately 1000 to 3500 g/mol [13]. Based on Pfeiffer’s study [14], the molecular mass of these four fractions is continuous in asphalt. Furthermore, there is no clear boundary between them, and together, they form a stable colloidal structure. Considering this system, asphaltenes micelles are the dispersed phase stabilized by Resins, whereas Aromatics and Saturates serve as the continuous phase in the continuous matrix [15].

Controlling the macro-mechanical properties of the asphalt by regulating the fractions is currently being performed by many researchers, and one successful example is the rejuvenation of asphalt [16]. Because asphalt is exposed to the natural environment for a long time, the saturated and aromatic fractions in the asphalt are gradually lost under extreme temperature cycling, tire pressure, and rainfall. Furthermore, the deposition of resins may also transform it into asphaltene [17], resulting in hardening and deformation of the asphalt. This is reflected in the loss of mechanical properties such as crack resistance, fatigue resistance, and ductility on a macroscopic scale [18]. By artificially supplementing aging asphalt with maltenes, a portion of the asphaltene can be dissolved, and its proportion can be reduced, achieving a recovery of the external mechanical properties [19,20]. In addition, considering some cold regions such as Ontario and Canada, the addition of motor oil or petroleum-based oil to hard asphalt to configure soft asphalt for low-temperature applications is an economical and efficient solution [21]. However, the types and amount of fractions added in these methods are still based on experimental experience, and it is unclear whether the addition of new fractions changes properties other than those targeted [22]. For example, in the use of waste engine oil modified asphalt, there have been cases where additional magnesium and iron ions have promoted the oxidation of the asphalt, leading to increased aging [23,24].

In recent years, manufacturing asphalt rejuvenators from inexpensive light oils and recycled waste oils has become a hot research topic. A typical process for these rejuvenator productions is filtration, sorting, and blending. Owing to their low cost and wide availability, three laboratory products are expected to be commercialized: waste cooking oil (WCO) [25], waste bio-oil (WBO) [26], and waste engine oil (WEO) [19]. Rayhan et al. [27] have shown that WCO can rapidly recover the penetration, softening point, and ductility of aged asphalts. Hu [28] used WEO, a combination of WEO and furfural extraction oil (WEO+FEO), and a combination of WEO and epoxy resin (WEO+ER) to rejuvenate short-term aged asphalt. The results show that WEO-rejuvenated asphalt does not perform well in terms of moisture and fatigue resistance. Lekhaz et al. [29] tested the performance of a mixture of stone mastic asphalt (SMA) and WEO-rejuvenated asphalt, the results of which contradicted the previous results, i.e., WEO-rejuvenated asphalt concrete showed good moisture stability. Many studies of these three come to the almost uniform conclusion that they are all effective in restoring the performance of aged asphalt, especially in fatigue and low-temperature performance [30,31,32]. However, an obvious shortcoming of them is the lack of high temperature performance, i.e., rutting resistance [33,34]. Recently, Li et al. [35] comprehensively reviewed the research on using WCO and WEO as asphalt rejuvenators and concluded that both of them are effective in restoring the rutting resistance, fatigue, and low temperature properties of aged asphalt as long as they are dosed in appropriate amounts. They also point out that although both WCO and WEO have shortcomings, combining them to formulate new regenerants is expected to solve these shortcomings, and this is the direction of future research.

The characteristics of different types of waste-oil-rejuvenated asphalt differ significantly owing to the obvious differences in the source, composition, and refining technology of the recovered oil [36]. Haghshenas et al. [37] evaluated the effect of five regenerants (paraffinic oil, aromatic extracts, naphthenic oil, triglycerides/fatty acids, and tall oil) with different chemical compositions. Aromatic extracts had the most similar SARA structure to typical asphalt binders and had the best anti-aging performance. Triglyceride/fatty acid and tall oil did not perform well on low-temperature performance and cohesion after long-term aging due to excessive oxygen content and carbonyl and hydroxyl functions. Paraffinic and naphthenic that contain high saturate may create compatibility issues with asphalt binders. Ding et al. [38] concluded that C_20_H_42_ can significantly increase thermoreversible aging in the base binder; however, C_32_H_66_ and asphaltene additives did not produce a similar effect. Shariati et al. [39] proposed a hybrid bio-oil rejuvenator, which can revitalize the aged binder by simultaneously desorbing and peptizing aged binder molecules. Heterocyclic HY molecules (1-butyl-Piperidine and N-methyl-2-Pyrrolidone) in this hybrid bio-oil rejuvenator play an important role, which can effectively improve the resistance of revitalized binder to moisture-induced damages. Another study by Ding et al. [40] pointed out that residual crystalline waxes in WEO would reduce the strain rate of asphalt at low temperatures and increase the risk of cracking. A study by our team also showed that unfiltered metal residues in WEO will accelerate the secondary aging of recycled asphalt [41].

Preliminary studies [42,43,44] have been conducted to show that the asphaltene content affects the basic indices of penetration, softening point, Frass brittle point, etc. Among different structural fractions, asphaltenes display the lowest temperature susceptibility [45], and they significantly contribute to bitumen stiffness, rigidity, and elasticity [46,47]. Xin et al. [48] further investigated the effect of polycyclic aromatic compounds (PAC), a major fraction of asphaltene, on asphalt, and found that the elasticity and complex modulus of asphalt were reduced with increasing PAC. Lesueur [49] provides a detailed review of the effect of asphalt colloid structure on rheology and chemistry properties, concluding that, although the asphaltene content is small, it is the main cause of the high viscosity and non-Newtonian rheological properties of asphalt. Speight et al. [12] confirmed the role of resins as stabilizers for asphaltenes, which would precipitate from the oily bitumen components without the resins. The potential relationships between the fraction composition and mechanical properties of asphalt found in previous studies are collated in Table 1. Most of these studies just focused on the relationship between asphaltene and mechanical properties, and the selected indicators were usually simple indexes such as stiffness, ductility, and elasticity [46,50,51,52]. In addition, studies on the complex rheological properties of binders usually consider asphalt as a single-fraction material [53,54,55], and few studies have investigated the effect of SARA fraction on rheological properties. There are significant differences in the physicochemical properties between SARA fractions, which have a significant impact on the rheological properties of asphalt binders. Therefore, it is difficult to reveal the rheological nature of asphalt binders without a full understanding of the effects of each fraction on asphalt [56].

A lack of efficient fraction separation techniques is a major impediment to studying the impact of single fractions [4]. Conventional SARA separation methods (such as column chromatography (commonly known as Corbett method) [59], recommended by ASTM D2424 [60]), are time-consuming, have high solvent consumption, and only 1–2 g of asphalt can be separated in a single pass. Some new methods have also been developed by scholars to separate SARA fractions; nevertheless, they are limited by equipment and solvents, etc. It is difficult to obtain the scalability. Handel’s new method, for instance, can separate 10 g in a single pass [61]; nonetheless, this amount is insufficient to be used as an additive to modify asphalt. Thin-Layer Chromatography with Flame-Ionization Detection (TLC-FID), originally a means of analyzing crude oil composition, is now also used for SARA analysis of asphalt. It can accurately and quickly obtain SARA distribution in one-fifth of the test time of the Corbett method [19]. Although this method is still unable to separate a large mass of independent fractions, it can be used to identify many oils with significant differences in composition and add them to the asphalt to observe their effects on the mechanical properties and fraction distribution of asphalt. This can be used to study a single change in asphalt properties.

It would help to advance the research work on asphalt regeneration and modification for specific application environments if the effect of each fraction on the macroscopic mechanical properties of asphalt could be identified. However, it seems that this work is currently limited by the lack of efficient methods for quantitative identification of fractions and uniform oil sources of asphalt, and no clear and uniform conclusions seem to have been reached in this work. Therefore, this study aims to investigate the correlation between the distribution of fractions of asphalt and its mechanical indicators. To achieve this objective, seven oil-rejuvenators with hugely different fraction compositions are selected to be added to the control of an aged asphalt 50/70 and are subjected to secondary aging, resulting in 16 asphalt samples. Basic property tests (such as penetration, ductility, and softening point), general rheological tests (such as critical cracking at low temperature (*T_CR_*), fatigue life at medium temperature (*N_f_*), non-recoverable creep compliance at high temperature (*J*_*nr*3.2_), and SARA fraction distributions TLC-FID analyses are performed on these 16 samples derived from the same asphalt. Eventually, the correlations between the fraction distribution and these mechanical indices are investigated.

## 2. Materials and Methods

### 2.1. Raw Materials

#### 2.1.1. Asphalt Binders

In this study, an aged asphalt 50/70 was used as the control and was analyzed for the mechanical properties and SARA fraction composition before and after adding oil-rejuvenators with different fractions composition. This control asphalt was recycled from the upper layer of the Chengdu-Chongqing expressway of China, which was completed and opened to traffic in 1995. After being in service for approximately 15 years, its performance grade was reduced from PG82-10 to PG70-22. The original asphalt (OA) had a needle penetration of 63 dmm(0.1 mm), a softening point of 47 °C, a 10 °C ductility of 26 cm, and a 135 °C viscosity of 0.44 Pa·s. After aging, it had a needle penetration of 31 dmm, a softening point of 65 °C, a 10 °C ductility of 5 cm, and a 135 °C viscosity of 1.32 Pa·s.

Its basic properties in the original and aged stated are summarized, where the penetration, softening point, ductility, viscosity, and performance grade were tested in accordance with the standard ASTM D5 [62], ASTM D36 [63], ASTM D113 [64], ASTM D4402 [65], and ASTM D6373 [66], respectively.

#### 2.1.2. Oil-Rejuvenator

Table 2 shows the properties of all the seven oil-rejuvenators that can be divided into two categories according to base-oil types. One is the four bio-rejuvenators with raw materials such as waste edible oil, tung oil, biodiesel, and fish oil residue labeled Bio-1, Bio-2, Bio-3, and Bio-4, respectively. Many studies involving regeneration and aging have discussed the improvement of the rheological properties of aged asphalt using these four regenerants; nevertheless, few studies have analyzed their fraction compositions [23,67,68,69].

The other is the three petroleum-based regenerators separated at different temperatures during vacuum distillation. Light fraction oil is the product of the distillation temperature 200–220 °C, labeled Pio-L, and in its internal molecular weight composition, C_12–18_, C_6–12_, and C_18-_ account for approximately 14%, 85%, and 1%, respectively. Middle fraction oil is fractionated at 220–260 °C, labeled Pio-M, in which C_12–18_, C_6–12_, and C_18-_ account for 95%, 2%, and 3%, respectively. Heavy fraction oil is fractionated at 220–260 °C, labeled Pio-H, in which C_12–18_ and C_18-_ account for 33% and 65%, respectively. It has no C_6–18_.

In Table 2, the acid value, iodine value, and saponification value of the four bio-rejuvenators were determined by the standard methods provided by ISO 660 [70], ISO 3961 [71], and ISO 3657 [72], respectively. For all the seven rejuvenators, their density at 20 °C, Kinematic viscosity at 60 °C, and flash point were determined by the standard methods provided by ASTM D4052 [73], ASTM D7279 [74], and ASTM D56 [75], respectively.

Fractions composition differences between these selected seven oil-rejuvenators are significant based on the TLC-FID test, which will be introduced in detail in Section 3.1.

### 2.2. Preparation of Oil-Rejuvenated Asphalt

Using high-speed shearing, oil-rejuvenated asphalts were made. The aged asphalt binder was heated to approximately 60 °C above the softening point (i.e., approximately 130 °C) and mixed with the rejuvenator, employing a shear mixer at a speed of 4000 r/min for 15 min [35]. The mixing condition was determined by the viscosity of aged asphalt 50/70 which is around 1.15~1.35 Pa·s at this temperature. In this specific range, the asphalt could be easily mixed with these rejuvenators. In previous mixing attempts, it was found that if the temperature was higher than this condition, some of the lighter rejuvenators (e.g., Pio-L) tend to volatilize (produce large amounts of white smoke) and thus affect the quality of the recycled asphalt. If the temperature is too low, the viscosity of the asphalt will be too high, and it will be difficult to miscible with heavy rejuvenators.

### 2.3. Design of Experiments

**Figure 1:**
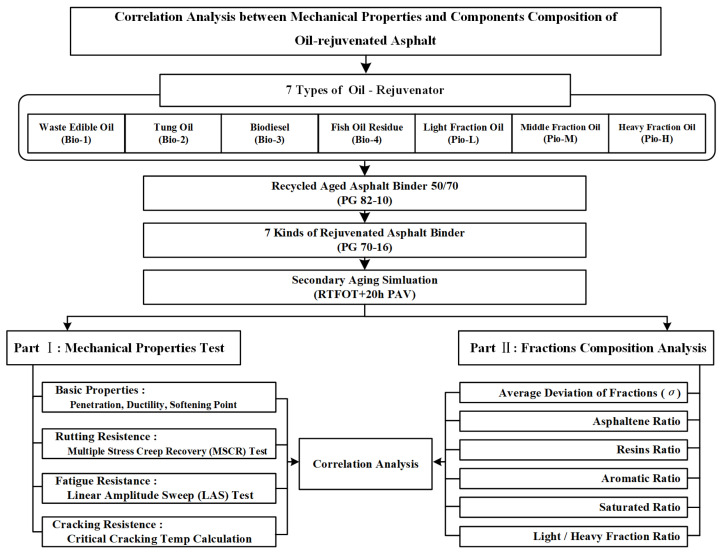
Schematic of the experimental test plan.

Figure 1 provides the experimental design of this study. First, the control aged asphalt 50/70 (RA) was rejuvenated using the seven oil-rejuvenators. Thereafter, these seven samples were subjected to secondary aging using the rolling film oven test (RTFOT) and 20-h pressurized aging vessel. Subsequently, all the asphalt samples were subjected to two parts of the test, i.e., mechanical property and fractions analysis tests. The mechanical property test includes multiple stress creep recovery (MSCR), linear amplitude scan (LAS), low-temperature critical cracking temperature calculation, and basic properties (penetration, softening point, ductility) tests. These were selected in many studies as a comprehensive set of indicators to evaluate the rutting, fatigue, and cracking resistance of the asphalt. Considering the fraction analysis tests, all the asphalt samples were separated and quantified for SARA (i.e., saturated, asphaltene, resinous, and aromatic) fractions using thin-layer chromatography with flame-ionization detection (TLC-FID, described in detail in Section 2.4.6). Finally, the correlation between the mechanical property indicators and fraction composition of these asphalts was analyzed separately.

### 2.4. Measurement and Characterization

#### 2.4.1. Basic Properties Test

Basic properties, including penetration at 25 °C, softening point, and ductility at 15 °C, were evaluated in accordance with the standard ASTM D5 [62], ASTM D36 [63], and ASTM D113 [64], respectively. Their duplicate tests were performed three times.

#### 2.4.2. Performance Grade (PG)

The temperature PG of all asphalt samples can be estimated using the dynamic shear rheological (DSR) and blending beam rheological (BBR) tests based on the AASHTO M320 [76] method. The DSR used in this study is the Discover HR-3 DSR manufactured by TA INSTRUMENT, and the BBR is the TE-BBR provided by CANNONTE.

#### 2.4.3. Low-Temperature Cracking Resistance Test

The critical cracking temperature (*T_CR_*) calculation is employed to evaluate the anti-cracking performance of oil-rejuvenated asphalts at low temperatures. *T_CR_* is a non-strength test index determined in the stiffness modulus data, while the stiffness modulus data are obtained from the extended bending beam rheological test [1]. This method can better reflect the ultimate performance of the asphalt at low temperatures than the PG test. It also has a high correlation with the actual pavement cracking [77].

The first step to calculate the *T_CR_* is to obtain the low-temperature stress *σ(ξ)* of the asphalt based on the continuous temperature decrease. This was calculated using the basic creep compliance *J(t)* data obtained from the asphalt binder by the BBR test. The specific steps for calculating the low-temperature stress *σ(ξ)* are based on AASHTO R49 [78] and the study by Roy and Hesp [77].

**Figure 2:**
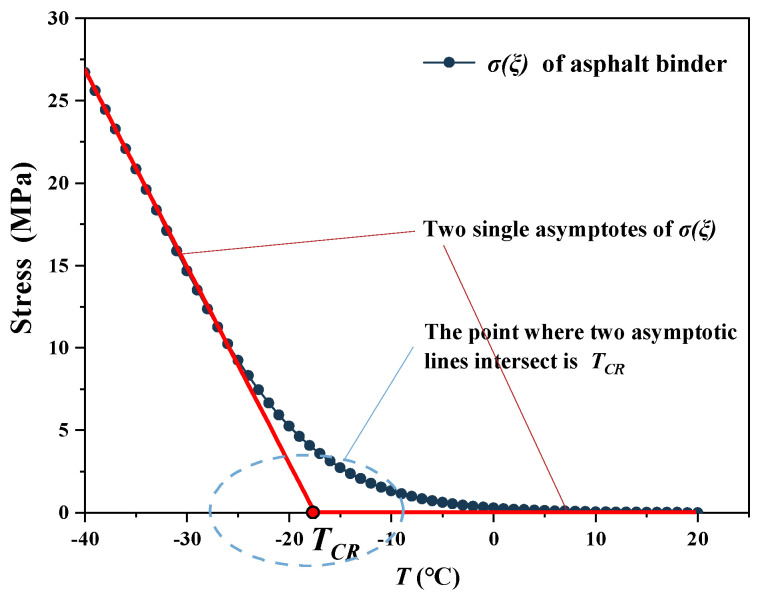
Temperature stress and TCR calculation according to the SAP theory [54].

The second step is to find the *T_CR_* in the curve of the *σ(ξ)* based on the theory of single asymptote procedure (SAP), proposed by Shony et al. [79] Figure 2 shows the change in the *σ(ξ)* as the temperature decreases. The temperature stress first increases gradually and then increases rapidly toward the end of the curve. The thermal stress limit is described using the asymptotes at the beginning and end of the thermal stress curve. The point where two asymptotic lines intersect is the *T_CR_*, where the curvature changes the fastest and cracks are most likely to occur. In this study, *T_CR_* is used as an indicator to assess the effect of oil-rejuvenators on the low-temperature crack resistance of asphalt. Smaller values of the *T_CR_* indicate a better low-temperature cracking resistance, indicating that the asphalt can be used at low ambient temperatures without cracking due to thermal stress.

#### 2.4.4. Linear Amplitude Sweep (LAS) Test

The LAS test was employed to evaluate the anti-fatigue performance of all the asphalt samples, and for each sample, two duplicates are tested. The LAS test can better simulate the loss development of asphalt under repeated loads than the PG test [80]. The LAS test includes two steps. The first step is frequency scanning at 0.1% strain in the frequency range of 0.1–30 Hz to determine parameters α and B in Equation (1). The second step is linear amplitude scanning, where a round of oscillatory load cycles with linearly increasing amplitudes (from 0.1% to 30%) is conducted at a constant frequency (10 Hz) to generate the accelerated fatigue damage. The viscoelastic continuous damage theory VECD (viscoelastic continuous damage) is used to determine parameter *A_35_* in Equation (1). The test method is based on AASHTO TP 101-12 [81], and a larger *N_f_* indicates a better fatigue resistance. The asphalt fatigue failure life (*N_f_*) is computed using Equation (1):

(1) $$N_{f}=A_{35}\cdot \gamma_{max}{}^{-B}$$

where $\gamma_{max}$ is the maximum expected asphalt strain for a given pavement structure, percent; *B* is equal to 2α, no unit; and *N_f_* represents the number of loading cycles before failure.

#### 2.4.5. Multiple Stress Creep Recovery (MSCR) Test

The MSCR test is employed to investigate the anti-rutting performance of all the asphalt samples, and the test method is based on ASTM D7405 [82]. Considering each sample, three duplicates are tested. The MSCR test has a better correlation with the anti-rutting performance of the asphalt compared to the rutting factor ($G^{*}/\sin\delta$). Therefore, it has gradually become a main method for identifying the high temperature performance of asphalts in the experiment system of Superpave [83]. For each asphalt sample, the MSCR test is performed at its performance grade (PG) high temperature. First, the specimen is loaded at constant creep stress for a 1-s length of time creep and followed with a zero-stress recovery of a 9-s length of time. Second, 20 creep and recovery cycles are performed at creep stress of 0.1 kPa. The first 10 cycles are for conditioning the specimen. The second ten cycles were designated as cycles from N = 1 to 10 and were employed for data collection and analysis. Thereafter, ten creep and recovery cycles are performed at creep stress of 3.2 kPa. The non-recoverable creep compliance measured at 3.2 kPa (*J*_*nr*3.2_) is employed as an assessment of the endurance of the bitumen to permanent distortion under repeated loading state, and a smaller *J*_*nr*3.2_ value represents a better rutting resistance [23].

#### 2.4.6. Fraction Analysis of the Asphalt

**Figure 3:**
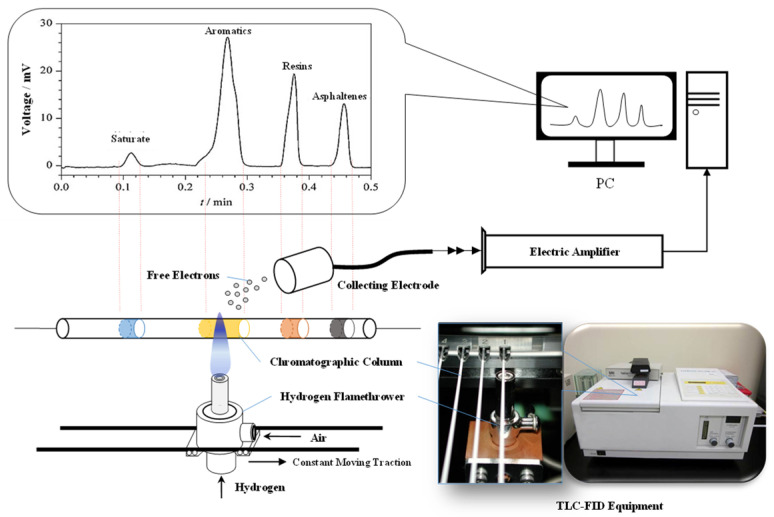
Test procedure and principle of the TLC-FID [19].

Compositional harmonic and compatibility theories are the most recognized theories regarding the asphalt aging phenomena [84]. Reduction in the proportion of the light fraction or increase in the heavy fraction of asphalt is considered the basis for asphalt aging by both theories [85]. Many studies attempt to justify the change in the asphalt properties by investigating the fractions changes before and after aging; however, the separation and quantification of fractions have always been very difficult tasks. Common fractions analysis methods include Fourier transform infrared reflection (FTIR) [86] and solvent precipitation methods (introduced in standard ASTM D4242 [60]). The former has a good testing efficiency; nonetheless, it is difficult to quantify the composition of the fractions. Moreover, the latter can obtain the exact amount; nevertheless, the process is complex, time-consuming, and lacks reproducibility. In our previous study, an efficient quantitative analysis method of asphalt fractions, TLC-FID, was introduced. This was derived from a chromatographic method used in the petrochemical industry. The procedure and principle of the TLC-FID are illustrated in Figure 3. A constructed chromatographic column that leverages the different diffusion heights of the four fractions of the asphalt in a toluene solution is scorched, during which the intensity of the electrons emitted by each fraction at the point of aggregation is recorded and converted into the amount of this fraction [19].

### 2.5. Dosage of Oil-Rejuvenators

**Figure 4:**
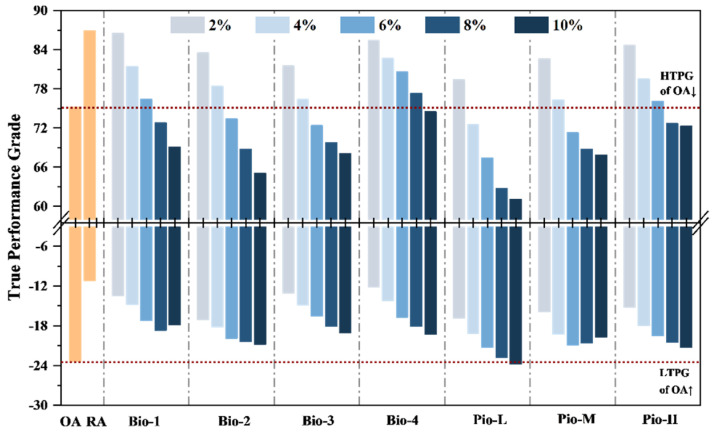
Relationship between the dosages and true performance grade of the seven oil-rejuvenators.

To ensure the comparability between the asphalt samples, the dosage of each rejuvenator was determined following the rule: under the selected dosage, the rejuvenator should restore the performance grade (PG) of the asphalt closer to its original status (i.e., the PG of OA) as much as possible. Therefore, a series of pre-tests were designed to characterize the relationship between the dosage of each rejuvenator and the PG of the recycled asphalt. The dosages of 2%, 4%, 6%, 8%, and 10% were used. The results are shown in Figure 4.

As shown in Figure 4, all the seven oil-rejuvenators are easy to recover the high-temperature PG (HTPG) of the RA to the level of OA (PG70-XX), where the critical dosages for the seven rejuvenators from Bio-1 to Pio-L are approximately 6%, 5%, 4%, 9%, 3%, 4%, and 6%, respectively. Once the dosages exceed the critical value, the HTPG of the rejuvenated asphalts will be worse than that of the OA. Considering the recovery effect of the low-temperature PG (LTPG), there are bottlenecks in these rejuvenators. When the dosage reached a certain value, the LTPG found it difficult to be further optimized or even deteriorate. No oil-rejuvenator could recover this index to the original level, except for 10% Pio-L. Obviously, there was no appropriate dosage for each oil-rejuvenator that could restore both the HTPG and LTPG of the RA to the original level simultaneously.

Considering most studies that focused on rejuvenated asphalts, a lack of high temperature performance has always been criticized [20,87]. Regarding this case, the consistency of the HTPG is prioritized, and the LTPG is in the same classification as much as possible. Therefore, the dosages of Bio-1, Bio-2, Bio-3, Bio-4, Pio-L, Pio-M, Pio-H were determined as 6%, 5%, 4%, 9%, 3%, 4%, and 6%, respectively, where the PG of all rejuvenated asphalt was PG 70-16.

## 3. Test Results

### 3.1. SARA Fractions Analysis

**Figure 5:**
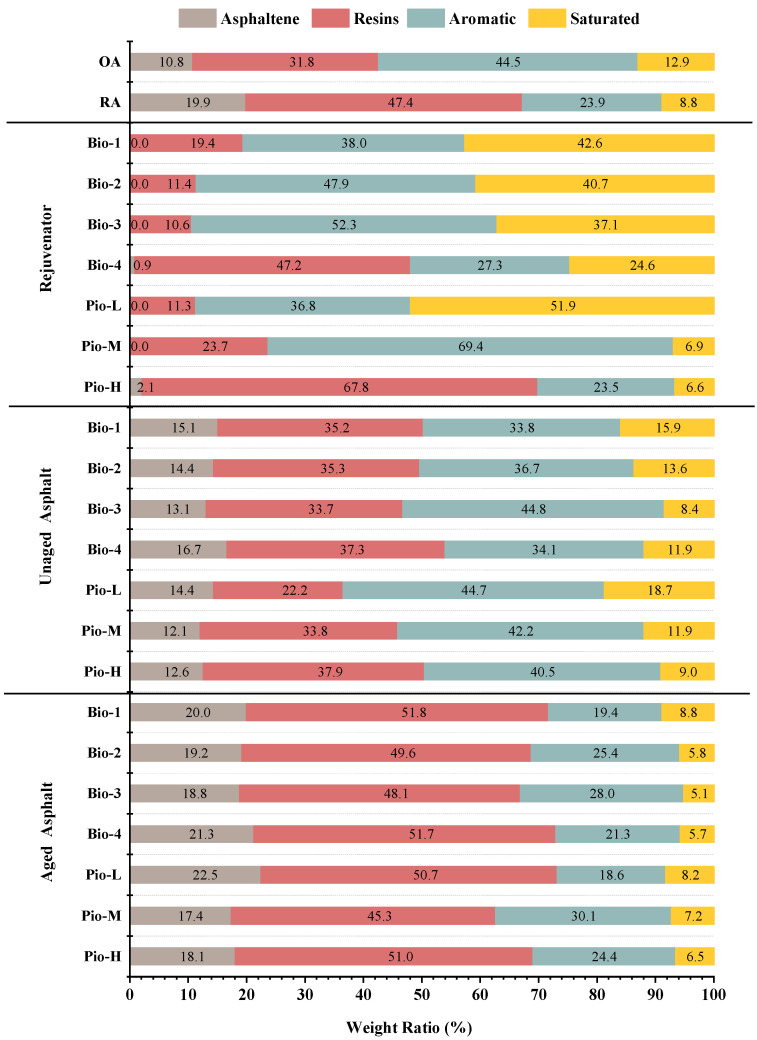
SARA fractions analysis of all the asphalt samples.

Figure 5 shows the SARA fractions results of all the samples. The top two bar graphs present the composition of the control asphalt OA and RA, where 24.7% (by mass ratio) of the aromatic and saturated (together called light fraction) were transferred to the asphaltene and resins (together known as heavy fraction) during the aging process. This is a typical aging process of asphalts [49].

The subsequent seven bars present the composition of the seven oil-rejuvenators. Considering Bio-1, Bio-2, and Bio-3, the proportion of aromatic increases sequentially, whereas the saturated gradually decreases. Regarding the Bio-4, light and heavy fractions are almost 50/50, and there is even 0.9% asphaltene in them. For the three petroleum-based rejuvenators, the ratio of the heavy fraction increased sequentially from 11.3 for Pio-L to 23.7 for Pio-M and to 69.9% for Pio-H.

The fraction composition of rejuvenated asphalt (unaged) is affected by its corresponding rejuvenator. Considering the unaged samples (Bio-1 to Bio-3), the proportion of the aromatic increases, whereas the saturated decreases, similar to their corresponding rejuvenators. Heavy fractions in the Bio-4 asphalt are also significantly higher than the other three bio-oil-rejuvenated asphalts.

After aging, the trends of the fraction structure change were the same for all the samples, where the ratio of asphaltenes to resins increased and the aromatic and saturated decreased. Many fraction composition characteristics of the aged bio-oil-rejuvenated asphalts inherit the characteristics associated with them in the unaged stage. For example, the ratio of resins decreased from Bio-3 to Bio-1. This inherited relationship can also be found in Pio-M and Pio-H. Nevertheless, Pio-L is an exception. Unaged Pio-L has the highest light fraction content of 63.4%. However, after the second aging, its ratio of heavy fraction became the highest. This could be attributed to its unstable colloidal structure.

Koots and Speights [12] indicated that a resin acts as a surfactant, creating a so-called soluble layer and helping to maintain the suspension of the asphaltene in the aromatic fraction of the dispersion system. If a binder has resins amounting to 50%, approximately 75% of them are needed to stabilize the asphaltene dispersion. Obviously, the ratio of resin to asphaltene in the Pio-L is poor, resulting in inadequate dispersion and suspension of the asphaltene. Therefore, even if a large number of aromatic fractions are supplemented as the dispersion system, they may simply mix with other fractions, without forming a stable and homogeneous colloidal structure. In this situation, the free aromatics may collect and oxidize to resin and asphaltene [19,41].

In Figure 5, none of the rejuvenators could reduce the RA’s asphaltene content to the level of the OA. Furthermore, the process for the resins content reduction appeared very difficult as well. This indicates that these rejuvenated asphalts produced by these rejuvenators are still essentially different from the OA, and aging has not been fully restored, although these rejuvenated asphalts have the same PG as the OA.

To characterize the degree of asphalt aging in its current state, a statistic, *average deviation of the fractions between the given asphalt and its original* (marked *σ*), was introduced in this study. Equation (2) gives the calculation of σ:

(2) $$\sigma =\sqrt{\frac{{\left(C_{\mathrm{Sat}}-C_{\mathrm{Sat}\;\mathrm{of}\;\mathrm{OA}}\right)}^{2}+{\left(C_{\mathrm{Asp}}-C_{\mathrm{Asp}\;\mathrm{of}\;\mathrm{OA}}\right)}^{2}+{\left(C_{\mathrm{Aro}}-C_{\mathrm{Aro}\;\mathrm{of}\;\mathrm{OA}}\right)}^{2}+{\left(C_{\mathrm{Res}}-C_{\mathrm{Res}\;\mathrm{of}\;\mathrm{OA}}\right)}^{2}}{df-1}}$$

where C_Sat_, C_Asp_, C_Aro_, and C_Res_ are the mass proportions of the four fractions (saturated, asphaltene, aromatic, and resins, respectively) of the asphalt samples in the current aging. C_Sat of OA_, C_Asp of OA_, C_Aro of OA_, and C_Res of OA_ are the mass proportions of the four fractions of this asphalt without any aging treatment (i.e., original asphalt). d*f* is the degree of freedom of the variable and takes the value of four.

**Figure 6:**
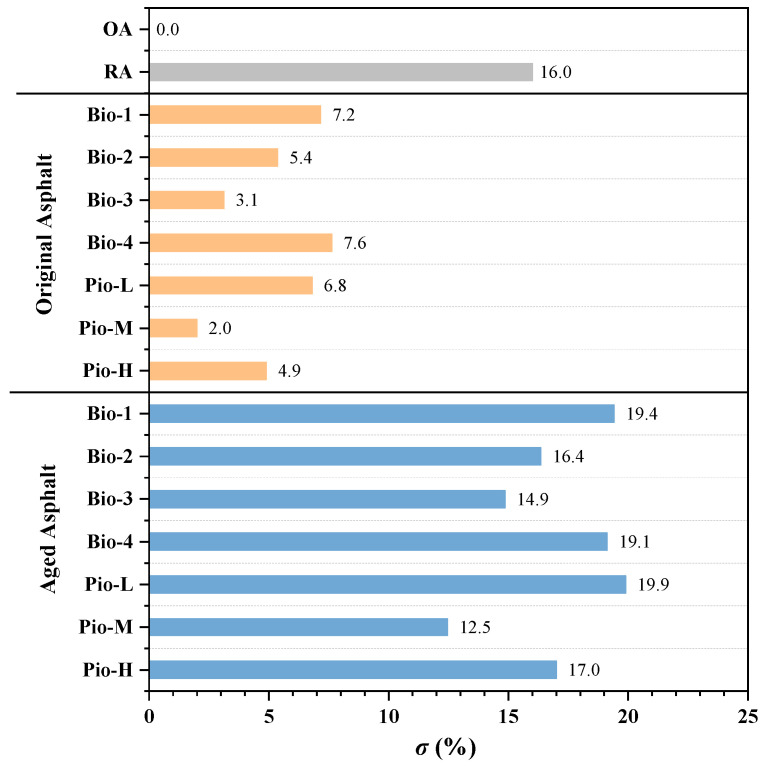
*σ* value of all the asphalt samples.

It is possible to quantify the degree of asphalt aging using *σ*, eliminating the need to describe the changes in the four variables (C_Sat_, C_Asp_, C_Aro_, and C_Res_) simultaneously. Particularly, *σ* reflects the average difference of the four fractions between the target asphalt and its original state, and it is used as an indicator to evaluate the degree of asphalt aging based on the composition. The smaller the *σ* value of asphalt, the less it differs from its original state, the less it deteriorates, and considering the rejuvenated asphalt, the better it recovers. The *σ* values of the seven oil-rejuvenated asphalt samples before and after the secondary aging are shown in Figure 6. Considering OA, *σ* is zero, and regarding the seven samples of the freshly regenerated asphalt, *σ* is ranked from smallest to largest as Pio-M, Bio-3, Pio-H, Bio-2, Pio-L, Bio-1, and Bio-4. This is also represented in the ranking of their regenerative effects (from best to worst). After the secondary aging, this ranking became Pio-M, Bio-3, Bio-2, Pio-H, Bio-4, Bio-1, and Pio-L, where *σ* values of Pio-H, Bio-4, Bio-1, Pio-L were greater than the RA. This indicates that the degree of these four rejuvenated asphalts after the secondary aging exceeded the ones before the regeneration. Characterizing the degree of asphalt aging, a detailed comparison between *σ* and the other mechanical indicators will be performed in Section 3.2, Section 3.3, Section 3.4, and Section 3.5.

### 3.2. Cracking Resistance

**Figure 7:**
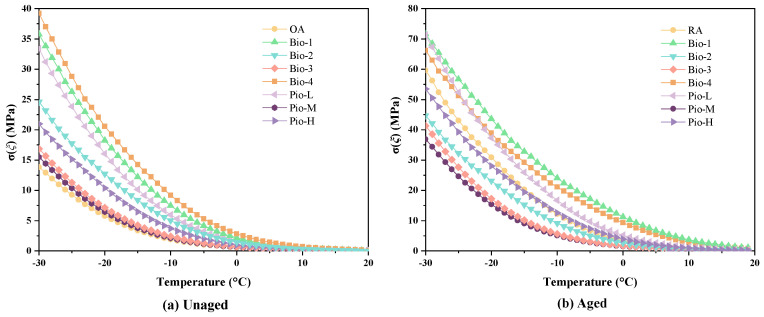
Low-temperature stress curve of (**a**) unaged asphalts (**b**) aged asphalts.

**Figure 8:**
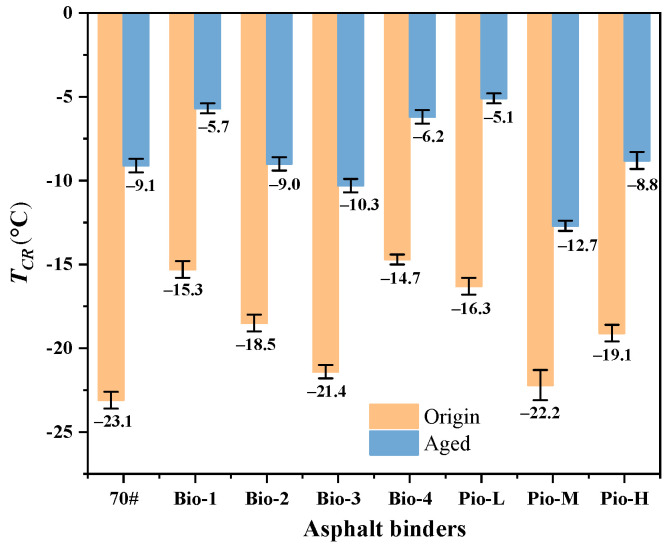
Critical cracking temperature (*T_CR_*) of all the asphalt samples.

Table 3 gives the results of the classical BBR tests, where $T_{s\;=\;300}$ and $T_{m\;=\;0.3}$ are the failure temperatures at stiffness equal to 300 MPa and *m*-value equal to 0.3, respectively. It difficult to accurately identify the low-temperature crack resistance of many samples referring the results, because multiple asphalts have the same LTPG. Therefore, the low temperature stress (*σ(ξ)*) and critical cracking temperature (*T_CR_*) were further calculated based on the classical BBR results for further performance grading, as shown in Figure 7 and Figure 8.

Figure 7 shows the curves of *σ(ξ)* of all the samples. Before the secondary aging, the curves of these rejuvenated asphalts are all above those of the OA, indicating that their temperature stresses are greater than those of the OA (Figure 7a). Correspondingly, the *T_CR_* values of the seven unaged rejuvenated asphalts are also higher than that of OA as shown in Figure 8. Considering the results of the *T_CR_*, the cracking resistance of the seven unaged samples and control at low temperatures can be ranked from best to worst as OA, Pio-M, Bio-3, Pio-H, Bio-2, Pio-L, Bio-1, and Bio-4. This seems to be generally consistent with the previous ranking of the degree of regeneration given by the *σ* values in Section 3.1. After the secondary aging, the *T_CR_* ranking changed to Pio-M, Bio-3, Bio-2, Pio-H, RA, Bio-4, Bio-1, and Pio-L, still consistent with the aged *σ* ranking. The *σ(ξ)* and *T_CR_* of the four samples, Pio-M, Bio-3, Bio-2, and Pio-L, are lower than the RA, indicating that their low-temperature crack resistance is better than that of the RA after secondary aging.

**Figure 9:**
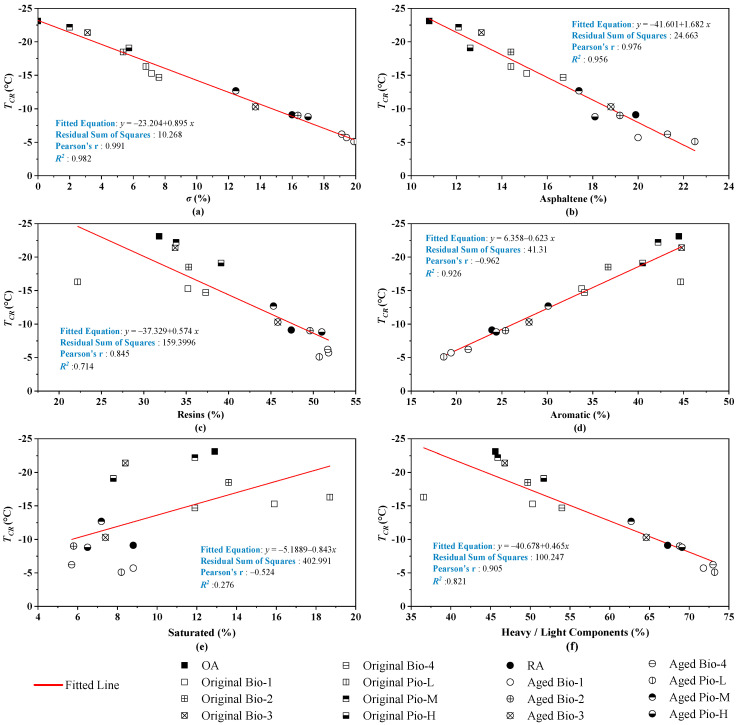
Correlation analysis between the *T_CR_* and fraction of (**a**) *σ*; (**b**) asphaltene; (**c**) resins; (**d**) aromatic; (**e**) saturated; (**f**) heavy/light.

There seems to be a correlation between the *T_CR_* and *σ*, where the larger the value of *σ* (the more the fractions change), the larger the value of the *T_CR_* (the worse the low-temperature performance of asphalt). To clarify the specific relationship between the fractions of these rejuvenated asphalts and their low temperature cracking performance, the correlation between the *T_CR_* and ***σ*** was investigated. The results of the one-dimensional correlation analysis between the ***σ*** values of all the 16 asphalt samples and their *T_CR_* are shown in Figure 9a, where the correlation coefficient (*R*^2^) is 0.982, which can be considered a strong correlation (*R*^2^ > 0.900). To further clarify which of the four fractions has a greater influence on the *T_CR_*, the correlations between this mechanical indicator and the proportion of asphaltene, resins, aromatic, saturated, and heavy fraction are investigated as shown in Figure 9. The correlation between the *T_CR_* and light fraction is the same as that with the heavy fraction because the light fraction is not an independent variable. The total content of the light and the heavy fraction is always 100%.

Considering the four fractions, *T_CR_* is strongly linearly correlated (*R*^2^ > 0.900) with asphaltene and aromatic ratio. The former is positively correlated (i.e., the higher the asphaltene content, the larger the *T_CR_*, and the worse the asphalt cracking resistance), and the latter is negatively correlated (i.e., the higher the aromatic content, the smaller the *T_CR_*, and the better the asphalt cracking resistance). Regarding the resins’ ratio, the *R*^2^ is 0.714, suggesting that it correlates (*R*^2^ in 0.6–0.9) with *T_CR_*. However, the *R*^2^ is only 0.276 (no correlation, *R*^2^ < 0.4) between the *T_CR_* and saturated ratio. This does not seem to have a significant effect on the low temperature performance. Li et al. [35] indicated that the correlation between low temperature performances and saturated fraction content of asphalt was not high, but these performances were strongly influenced by the ratio of asphaltene to aromatic fraction. Based on these findings, the key reason for the superior performance of the four rejuvenated asphalts (Bio-2, Bio-3, Pio-M, and Pio-L) over the RA is because they all have less asphaltene and are more aromatic than the RA. Similarly, the *T_CR_* of the seven unaged rejuvenated asphalts is worse than that of the OA because their asphaltene content was higher than that of the OA despite the dispersion and dissolution efforts.

### 3.3. Fatigue Resistance

**Figure 10:**
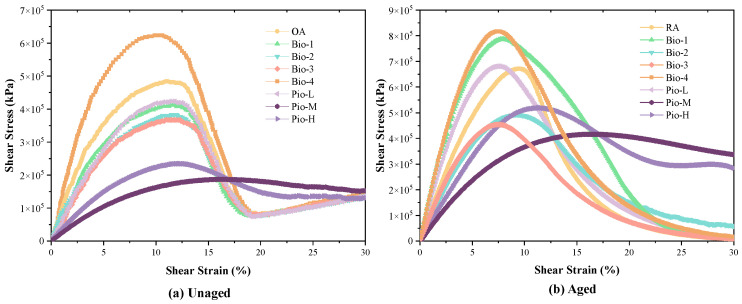
Shear strain versus shear stress curves of the LAS test of (**a**) unaged asphalts (**b**) aged asphalts.

The LAS test at 25 °C under 5% strain was applied to all 16 asphalt samples. The curves in Figure 10 are the results of the amplitude scan in the LAS test. This illustrates the relationship between shear stress and strain. There is a shear stress peak in every curve, which can be referred to as the yield stress, and the strain of it is known as the yield strain. The asphalt that possesses smaller yield stress and much yield strain has a better performance to adapt to the transformation in the repeated load. The shear strain–stress curve of the eight asphalt samples before aging can be divided into two categories. The first includes OA, Bio-1-4, and Pio-L, whose stresses first increase with strain and gradually decrease after reaching the yield stress. A stress rebound occurs at approximately 19% of the strain. Another category, including Pio-M and Pio-H, did not have a rebound, and their stresses gradually decayed after reaching the yield stress. After aging, the stress rebound phenomenon in the first category of the curve disappeared and was replaced by a constant and moderate stress drop. Nonetheless, another type of curve maintained the same basic shape before and after aging, except for the stress increase.

**Figure 11:**
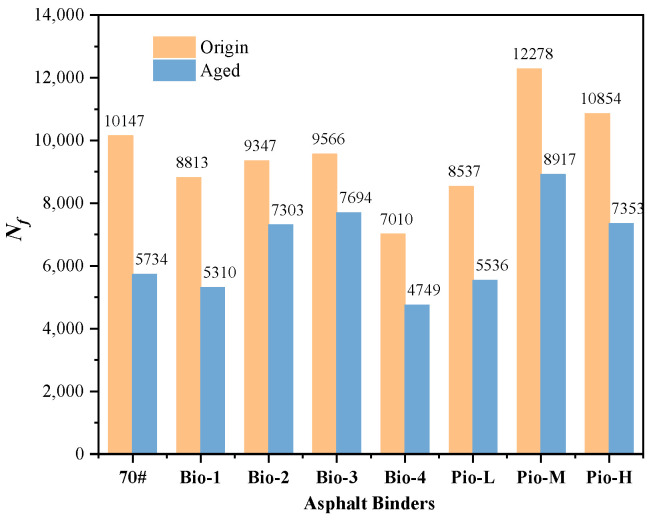
Fatigue failure life (*N_f_*) of all the asphalt samples.

The fatigue properties characterized by fatigue failure life (*N_f_*) of the unaged samples are ranked from best to worst as Pio-M, Pio-H, OA, Bio-3, Bio-2, Bio-1, Pio-L, and Bio-4, whereas the ranking changes to Pio-M, Bio-3, Pio-H, Bio-2, RA, Pio-L, Bio-1, and Bio-4 after secondary aging as shown in Figure 11. Pio-M and Pio-H (which performed well in the low-temperature test) continued to perform well in the phase, whereas Pio-L, Bio-1, and Bio-4 (which performed poorly in the previous test) continued to perform poorly in this segment.

**Figure 12:**
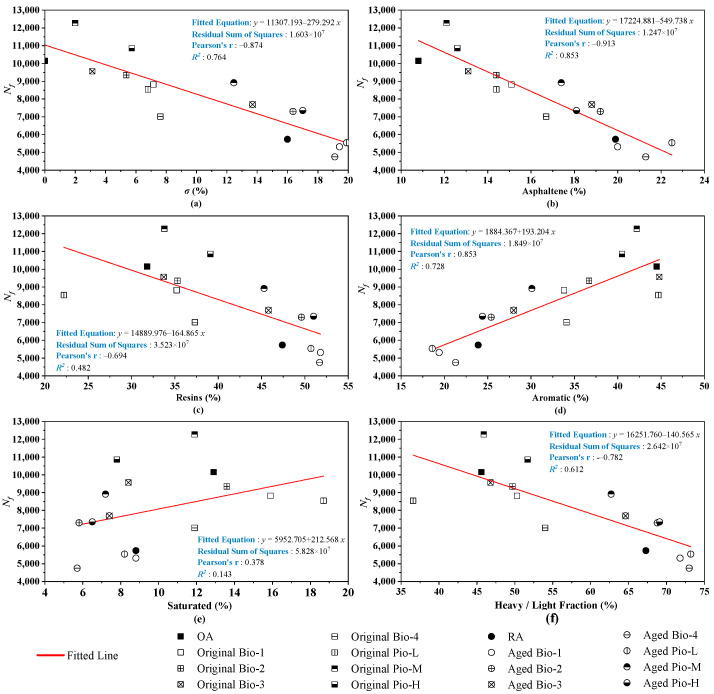
Correlation analysis between the *N_f_* and fraction indicators.(**a**) *σ*; (**b**) asphaltene; (**c**) resins; (**d**) aromatic; (**e**) saturated; (**f**) heavy/light.

Similar to the *T_CR_*, the correlations between *N_f_* and the six fraction indicators are analyzed and shown in Figure 12. Considering the six fraction indicators, *N_f_* had correlation coefficients greater than 0.6 with the *σ*, asphaltene, aromatic, and heavy/light fraction ratios. However, there was a weak correlation (*R*^2^ in 0.4–0.6) between the *N_f_* and resins’ ratio, and almost no correlation (*R*^2^ < 0.4) with the saturated ratio. The *R*^2^ between the *N_f_* and all the six fraction indicators is significantly smaller than that of the *T_CR_*. This may be because the fatigue performance of the oil-rejuvenated asphalt is the result of the four fractions harmonizing with each other, rather than being determined by one or two key fractions as in the case of the *T_CR_*.

### 3.4. Rutting Resistance

**Figure 13:**
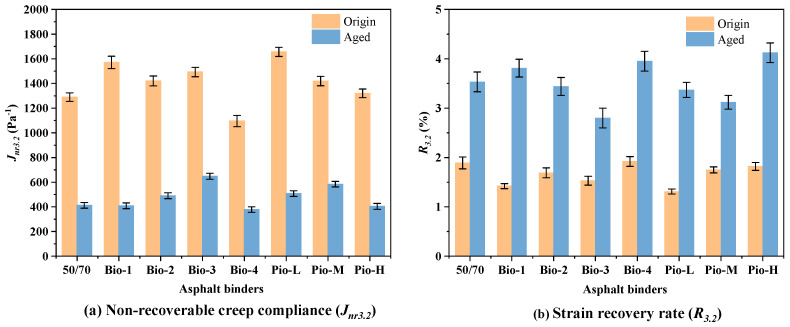
MSCR test results of all the asphalt samples.

All the asphalt samples were conducted using the MSCR test at the stress of 3.2 kPa. The test temperature for each sample is the high temperature with its performance grading without grade bumping. The two main indexes of this test, non-recoverable creep compliance (*J*_*nr*3.2_) and strain recovery rate (*R*_3.2_), are shown in Figure 13a,b. Asphalt with a good rutting resistance should have a small *J*_*nr*3.2_ and large *R*_3.2_.

The rutting resistance of these unaged asphalt samples is ranked (from best to worst) Bio-4, OA, Pio-H, Pio-M, Bio-3, Bio-2, Bio-1, and Pio-L based on the *R*_3.2_ and *J*_*nr*3.2_. After aging, this ranking changes to Bio-4, Pio-H, Bio-1, RA, Bio-2, Pio-L, Pio-M, and Bio-3. The ranking here is almost opposite to the previous ranking of the crack and fatigue resistances. Pio-M and Bio-3 have perfect *N_f_* and *T_CR_*; nonetheless, they exhibit the worst irrecoverable flexibility and strain recovery in the MSCR test. Moreover, Pio-L, Bio4, and Bio-1, which previously performed poorly, exhibit good high-temperature rutting resistance.

**Figure 14:**
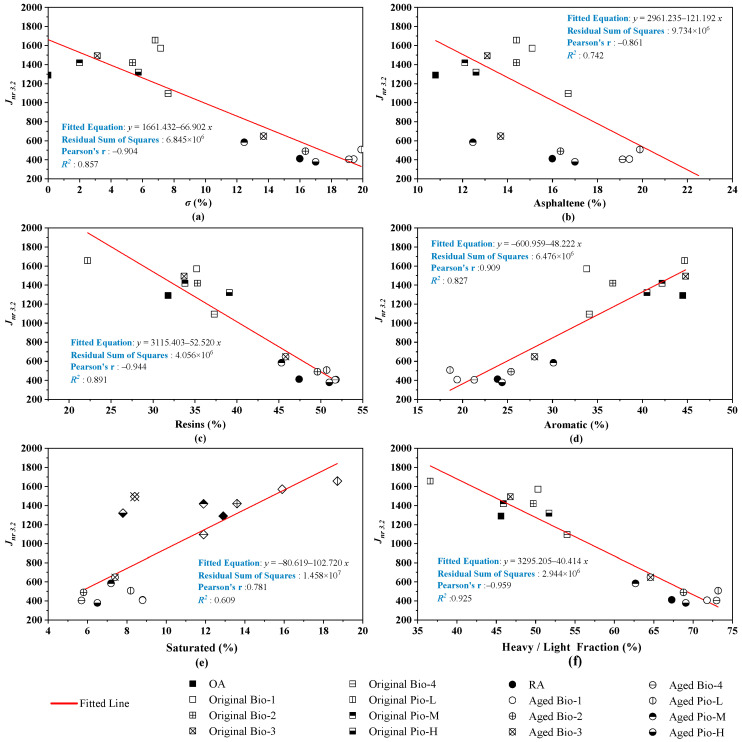
Correlation analysis between the *J*_*nr*3.2_ and fraction indicators. (**a**) *σ*; (**b**) asphaltene; (**c**)resins; (**d**) aromatic; (**e**) saturated; (**f**) heavy/light.

The correlation analysis of *J*_*nr*3.2_ with the six fraction indicators is shown in Figure 14, where all the six correlation coefficients are greater than 0.6. Particularly, Resins, which were not previously closely related to fatigue and low-temperature crack resistance, maintain a good correlation with *J*_*nr*3.2_ (*R*^2^ = 0.891). Furthermore, the heavy fraction strongly correlates with *J*_*nr*3.2_ (*R*^2^ = 0.925) because resins make up a large proportion of the heavy fraction. This indicates that the heavy fractions play a major role in the rutting resistance of these rejuvenated asphalts.

### 3.5. Penetration, Ductility, and Softening Point

**Figure 15:**
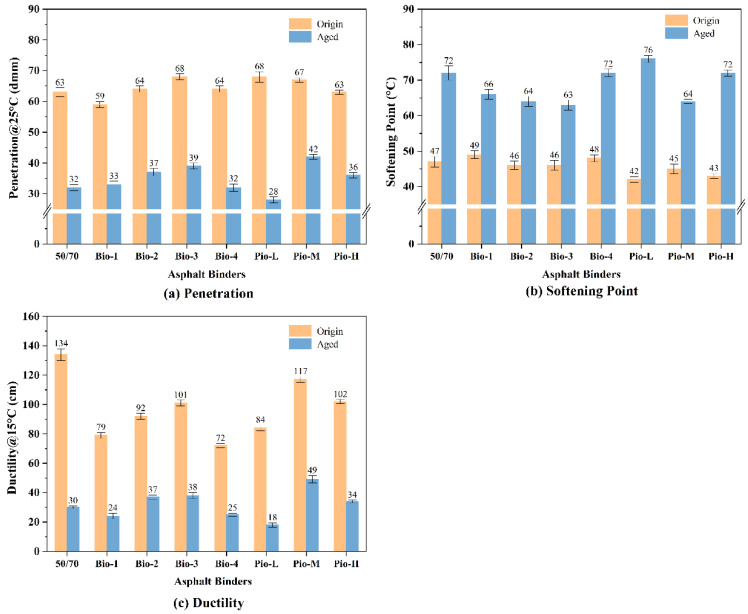
Basic empirical properties of all the asphalt samples: (**a**) penetration (**b**) softening point (**c**) ductility.

The test result of the three basic empirical properties: penetration, softening point, and ductility, are shown in Figure 15. Considering the unaged samples, their penetration and softening point were similar to those of the OA, and the differences were not more than 6%. However, regarding 15 °C ductility, none of the rejuvenated asphalts could be recovered to the level of the OA. In addition, there are significant differences in the ductility values of different rejuvenated asphalt. According to many asphalt material standards, ductility at 15 °C, greater than 100 cm, is a mandatory requirement for asphalt 50/70; however, only Pio-M, Pio-H, and Bio-3 meet this requirement. Based on the magnitude of the ductility, the recovery effects of the seven unaged samples can be ranked as Pio-M, Pio-H, Bio-3, Bio-2, Pio-L, Bio-1, and Pio-4 (from best to worst). It can be confirmed that oil regenerators easily completely recover the penetration and softening points; nevertheless, it is difficult for ductility.

After secondary aging, the penetration and ductility of all the asphalt samples decreased, softening point increased, and there were significant differences among the samples. The ranking of the penetration from the largest to smallest is Pio-M, Bio-3, Bio-2, Pio-H, Bio-1, RA, Bio-4, and Pio-L, which reflects the degree of hardening of these binders. Considering the 15 °C ductility, the ranking from the best to worst is Pio-M, Bio-3, Bio-2, Pio-H, RA, Bio-4, Bio-1, and Pio-L, reflecting the deformability of these asphalts. These two rankings are almost identical, suggesting a correlation between the reduced deformability and hardening of these asphalt binders. This could be due to the changes in their internal fractions.

**Figure 16:**
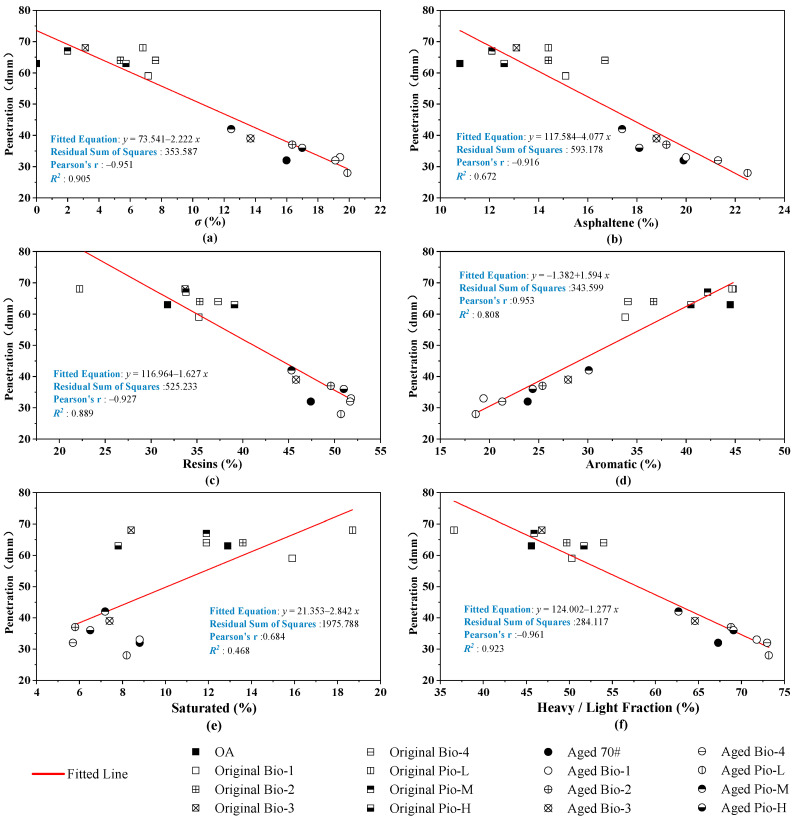
Correlation analysis between the penetration and fraction indicators. (**a**) *σ*; (**b**) asphaltene; (**c**)resins; (**d**) aromatic; (**e**) saturated; (**f**) heavy/light.

**Figure 17:**
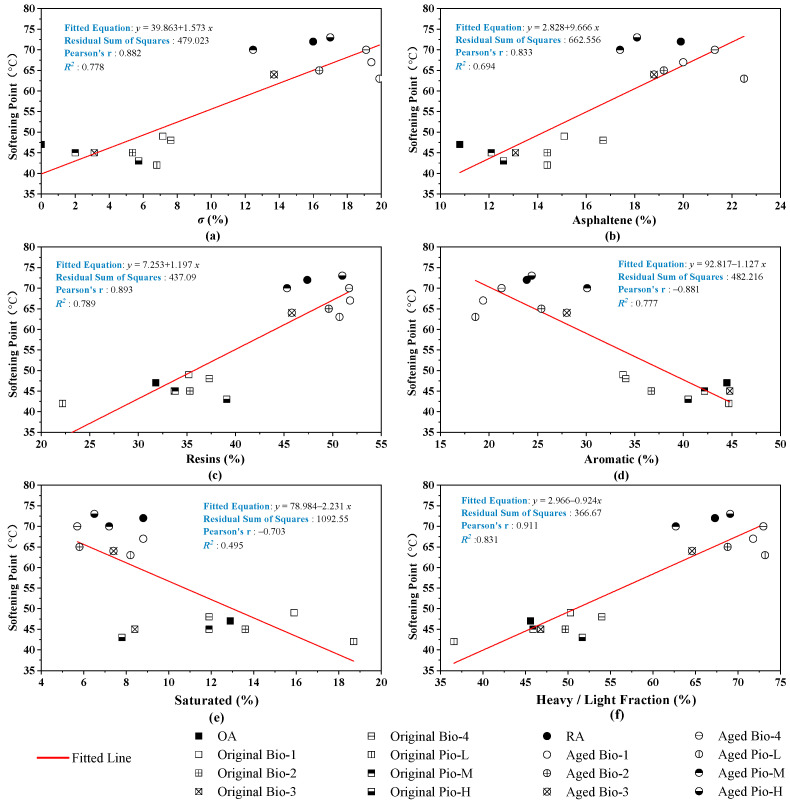
Correlation analysis between the softening point and fraction indicators. (**a**) *σ*; (**b**) asphaltene; (**c**)resins; (**d**) aromatic; (**e**) saturated; (**f**) heavy/light.

**Figure 18:**
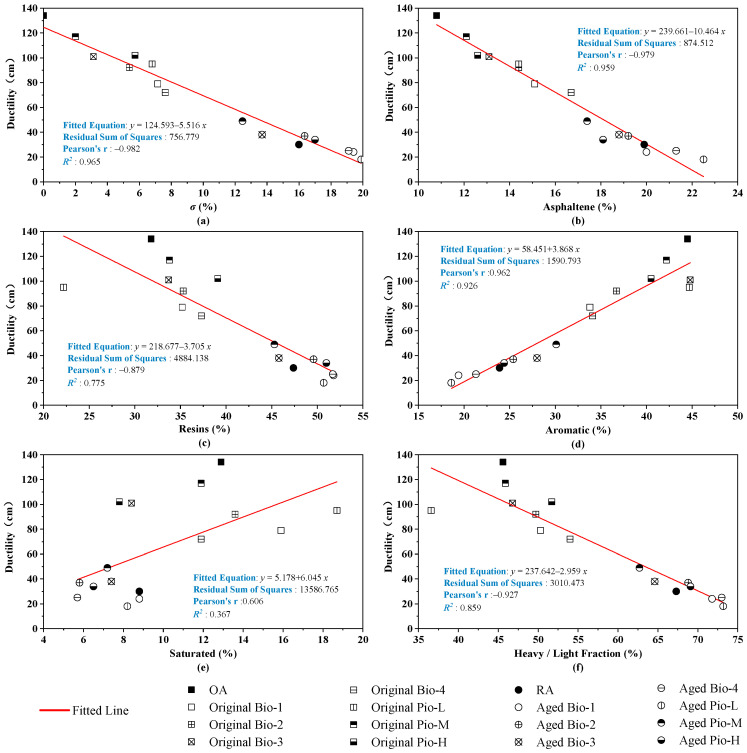
Correlation analysis between ductility and fraction indicators. (**a**) *σ*; (**b**) asphaltene; (**c**)resins; (**d**) aromatic; (**e**) saturated; (**f**) heavy/light.

The correlations of the three basic empirical indicators with the six fraction indicators are analyzed in Figure 16, Figure 17 and Figure 18. Considering these correlations, ductility and penetration generally have large *R*^2^ with these fraction indicators, where the *R*^2^ are greater than 0.9 of penetration with the ***σ*** and light/heavy fraction and an *R*^2^ even greater than 0.95 in ductility of the *σ* and asphaltene. However, the correlations between the softening point and these fraction indicators are not as close as the two previous basic mechanical indicators, where the correlation coefficients only range from 0.6 to 0.8.

## 4. Discussion

Table 4 summarizes all the rankings of the asphalt mechanical indicators for a systematic comparison, and some valuable conclusions can be drawn as follows.

Rankings of most asphalt samples before and after aging did not change significantly, generally within one to two places; however, the asphalt 50/70 was an exception. Before aging, it (OA) had the top performance of ductility, low-temperature crack, second rutting, and the third fatigue resistances; nevertheless, after aging (RA), they regressed to approximately ranking four or five. The OA has an absolute advantage, considering the low temperature cracking resistance and ductility; nonetheless, after aging, they could no longer be recovered regardless of the type of oil-rejuvenator used. After the second aging, if the benchmark of the aged asphalt 50/70 is still used for judgment, the comparison shows that only Pio-H and Bio-2 are better than the RA, considering the overall performance. Bio-1 and Bio-4 are excellent in rutting performance; nonetheless, they lag in cracking resistance, fatigue resistance, and ductility. By contrast, Pio-M and Bio-3 are superior to Bio-1 and Bio-4, considering the crack and fatigue resistances. They also performed poorly in the rutting resistance. Pio-L is almost always far behind the RA in all performance, considering only *T_CR_*, *N_f_*, *J*_*nr*3.2_, and ductility because it is neither good nor bad for the penetration and softening point.

A detailed comparison revealed that the rejuvenated asphalt ranked almost the same for the low-temperature crack resistance, fatigue resistance, and ductility, regardless of the degree of aging. This is similar to the penetration, softening point, and high-temperature rutting resistance; however, it is opposite to the three previous indicators. This may be related to how these tests are performed, which can be broadly classified into two categories. The first category (*T_CR_, N_f_,* and ductility) examines the ability of the asphalt to adapt to deformation at medium or low temperatures [53,88,89], whereas the second category (*J*_*nr*3.2_, *R*_3.2_, penetration, and softening point) examines the ability of the asphalt to resist or recover deformation at high and medium temperatures [20]. Therefore, the ranking of the indicators obtained from the same category of the test may have some similarities.

Furthermore, there are large internal differences in the mechanical indicator among the three petroleum-based rejuvenated asphalts. Pio-M has an excellent deformation ability, Pio-L seems to harden severely and performs poorly in all aspects, whereas Pio-H has a more balanced ability (not particularly outstanding or particularly poor). All three of them were products of petroleum vacuum distillation; nonetheless, the different composition of the rejuvenator fractions leads to significant differences in the properties of the recovered binders [41]. Pio-L and Bio-1 are the two rejuvenators with the highest saturated fraction. They also brought in a large amount of saturated fraction into the aged asphalt, which seems to add a large amount of lighter fraction to the asphalt. However, many studies have reported that rejuvenators containing high amounts of saturates will cause compatibility issues with asphalt binders in long-term aging and may aggravate the imbalance of the asphalt colloidal structure [37,49]. More importantly, aging often occurs in functional groups with high oxygen content (e.g., carboxyl, aldehyde), which are enriched in saturated fractions [90]. Therefore, the mechanical properties of Pio-L and Bio-1 decay rapidly in secondary aging. However, since the aromatic fraction does not contain aging groups [91], Pio-M and Bio-3, which contain more than 50% aromatic fractions, performed well in long-term aging after the addition of aged asphalt.

Table 5 summarizes the correlation coefficients between the fraction composition and six indicators that can fully characterize the mechanical properties of the asphalt, from which a lot of valuable information can also be obtained.

The *T_CR_* and ductility, which characterize the deformation performance of asphalts at low and medium temperatures, are strongly correlated with the asphaltene ratio, aromatic ratio, and *σ* (*R*^2^ > 0.900). A comparative analysis of Figure 5, Figure 8 and Figure 15c shows that, considering any sample of the asphalt binder, the proportion of asphaltenes is usually low (unaged binder < 15%, aged binder < 22%) whereas aromatic is large (unaged binder in 30–45%, aged binder in 20–30%), both of which profoundly affect the ability of the asphalt to adapt to deformation [23]. Considering the asphalt samples with excellent *T_CR_* and ductility, their ratio of aromatic to asphaltene is large, and because the ratio becomes smaller, these two mechanical indicators become progressively worse. Based on the explanation of the colloidal structure, because the saturated and aromatic contents in the dispersion medium decrease and the content of the protective substance resins and asphaltene in the dispersion phase increases, the asphalt changes from sol structure to sol-gel structure or even gel structure. Meanwhile, the asphalt becomes increasingly hard, and its deformation properties become worse [92,93]. The results of the correlation coefficients in Table 5 show that asphaltene and aromatic are the most important fractions of the dispersed phase and dispersion medium, respectively. Moreover, they play a decisive role in the deformation adaptability of asphalt. Based on the above reasons, considering the regeneration of the RA in this study, the *T_CR_* and ductility of these regenerated asphalts are hardly comparable to the OA because none of the oil-rejuvenators can reduce asphaltene and raise the aromatic score to the level of OA. This also leads to asphalts that deviate more from the OA (i.e., the larger the ***σ*** possessed, the worse their *T_CR_* and ductility), indicating the strong correlation between ***σ*** and these two mechanical indicators.

Moreover, *J*_*nr*3.2_ and penetration are strongly correlated (*R*^2^ > 0.900) with the heavy composition. Meanwhile, resins (the main component in the heavy fractions) also have a high correlation with these two mechanical indicators (*R*^2^ = 0.891 for *J*_*nr*3.2_ and *R*^2^ = 0.879 for penetration). Its proportion in the unaged samples is approximately 30%, and this can increase to 50% after aging. In addition, considering the *N_f_* and softening point, their correlations with these fraction indicators show a balanced relationship, with no significant strong correlation for one or two of them. This indicates the reconciliation effect between the four fractions that jointly affect these two mechanical properties.

In summary, although asphaltene is the smallest fraction of asphalt, it has a controlling effect on all mechanical performance of asphalt. Aromatic fraction content is closely related to the low temperature strain rate of asphalt. The larger its proportion, the stronger is asphalt’s low-temperature cracking resistance and resistance to aging. Saturated fraction does not seem to be correlated with most mechanical performance, but its percentage should not be too high, otherwise, it will lead to accelerated aging of asphalt. In addition, the coordination between the four fractions determines the fatigue resistance and softening point of the asphalt. They do not seem to be influenced much by a single fraction as much as the other four mechanical indicators.

## 5. Conclusions

This study investigates the correlation between the distribution of asphalt fractions and mechanical indicators, especially the current commonly used rheological indicators, of 16 asphalt samples originating from the same asphalt; nonetheless, they have widely varying fraction compositions, and the following important conclusions are drawn:

- A new indicator, *average deviation of fractions between the given asphalt and its original* (marked ***σ***) is proposed to characterize the degree of asphalt aging based on the variations of SARA fractional composition. It normalizes the four simultaneous change indicators (percentage of SARA fractions) during asphalt aging into one indicator. This new indicator has a strong correlation with several mechanical performance indicators of asphalt. Experiments and statistical analysis show that it is strongly correlated (*R*^2^ > 0.90) with *T_CR_*, ductility, and penetration, has a good correlation (*R*^2^ > 0.85) with *J*_*nr*3.2_, and is correlated with (*R*^2^ > 0.75) fatigue life (*N_f_*) and softening point. ***σ*** provides a potential approach to further investigate the inner relationship between SARA fraction composition and its mechanical properties of asphalts;
- The correlation between asphalt SARA fractions and their several mechanical indicators is clarified. Asphaltene fraction content is strongly correlated (*R*^2^ > 0.95) with low temperature critical cracking temperature (*T_CR_*) and ductility. Aromatic fraction content is also strongly correlated (*R*^2^ > 0.92) with these two indicators, *T_CR_* and ductility. Heavy fraction (asphaltene and resins) content is strongly correlated with (*R*^2^ > 0.90) penetration and non-recoverable creep compliance (*J*_*nr*3.2_ from MSCR test). Resins also have a good correlation (*R*^2^ > 0.88) with penetration and non-recoverable creep compliance (*J*_*nr*3.2_). Saturated fraction is not significantly correlated with these mechanical indicators (all *R*^2^ < 0.61);
- Effects of some SARA fractions in asphalt regeneration are evaluated. Asphaltene has a controlling effect on all the mechanical performance of asphalt. The larger the aromatic proportion, the asphalt’s low-temperature cracking resistance and resistance to aging ability are also stronger. The content of saturated fraction should not be too high, otherwise, it may accelerate aging of asphalt. In addition, all the oil-rejuvenators can barely restore the *T_CR_* and ductility of the aged asphalt to their original levels because it is difficult to completely reduce the asphaltene content to the original level;
- More currently used mechanical properties indicators, especially those with complex rheology, can be examined for their correlation with fraction composition by the methods presented in this study. This type of work will provide a reference for the directional design of bitumen for applications in complex environments.

## Figures and Tables

**Table 1:** Effect of fractions on mechanical properties of asphalt in previous studies.

Studies	Fractions	Influence on Mechanical Properties
Corbett, L.W. [42]	Saturates and aromatics	Positive correlation with hardness, temperature susceptibility, softening point
Sultana and Bhasin [57]	Saturates and aromatics	Negative correlation with tensile strength
Loeber, L. et al. [46]	Asphaltenes	Positively correlated with G* and stiffness
Ghasemirad, A. et al. [47]	Asphaltenes	Positively correlated with stiffness, elasticity, and high-performance grade (PG)
Hofko, B. et al. [50]	Asphaltenes	Positively correlated with stiffness and elasticity
Fernandez et al. [51]	Asphaltenes and resins	Positively correlated with penetration and negative correlation with ductility
Cooper et al. [52]	Asphaltenes	Negative correlation with fracture resistance
Xin et al. [48]	PAC in Asphaltenes	Positively correlated with elasticity and complex modulus
Speight, J.G. [12]	Resins	It is a stabilizer of asphaltene
Petersen, J.C. [58]	All the four fractions	Durability
Redeliusa, P. et al. [43]	Asphaltenes and aromatics	Respectively positive and negative correlation with viscosity
Haibo D. [38]	Asphaltenes	No significant correlation with low temperature performance

Note G* is the complex modulus of asphalt binders.

**Table 2:** Properties of the seven oil-rejuvenators.

Raw Materials	Base Oil	Label	Acid Value (mg KOH/g)	Iodine Value (g I/100 g)	Saponification Value (mg KOH/g)	Density @20 °C (cm)	Kinematic Viscosity @60 °C (mm^2^/s)	Flash Point (°C)	Appearance
Waste edible oil	Bio-oil	Bio-1	≤7.1%	274	181	0.927	61.7	316	Brown, cloudy
Tung oil		Bio-2	≤0.4%	76	182	0.944	70.3	224	Yellow, transparent
Biodiesel		Bio-3	≤2.9%	143	192	0.965	68.6	251	Yellow, transparent
Fish oil residue		Bio-4	≤3.4%	173	198	0.994	100.5	210	Yellow, translucent
Light fraction oil	Petroleum extract	Pio-L	-	-	-	0.893	51.2	198	Yellow brown, translucent
Middle fraction oil		Pio-M	-	-	-	0.936	88.4	224	Colorless, transparent
Heavy fraction oil		Pio-H	-	-	-	1.016	152.4	240	Black, opaque

**Table 3:** Classical BBR test results of all the asphalt samples.

Asphalt	Aging State	Failure Temperature		LTPG
		$T_{s\;=\;300}\;(\text{°}C)$	$T_{m\;=\;0.3}\;(\text{°}C)$	
50/70	Unaged	−28.3	−24.9	PG XX-22
	Aged	−17.2	−14.5	PG XX-10
Bio-1	Unaged	−21.7	−17.8	PG XX-16
	Aged	−14.8	−10.4	PG XX-10
Bio-2	Unaged	−24.0	−21.1	PG XX-16
	Aged	−18.4	−13.6	PG XX-10
Bio-3	Unaged	−25.9	−22.2	PG XX-22
	Aged	−16.3	−14.6	PG XX-10
Bio-4	Unaged	−21.8	−16.9	PG XX-16
	Aged	−14.9	−11.2	PG XX-10
Pio-L	Unaged	−23.2	−17.7	PG XX-16
	Aged	−12.9	−9.7	PG XX-04
Pio-M	Unaged	−28.9	−24.0	PG XX-22
	Aged	−20.3	−16.9	PG XX-16
Pio-H	Unaged	−25.6	−21.5	PG XX-16
	Aged	−18.7	−13.6	PG XX-10

**Table 4:** Ranking of the mechanical indicators of all the asphalt samples.

Asphalt Samples	Raw Materials of Rejuvenator	Aging State	Ranking of Mechanical Indicators					
			Cracking Resistance (*T_CR_*)	Fatigue Resistance (*N_f_*)	Rutting Resistance (*J*_*nr*3.2_ & *R*_3.2_)	Penetration	Ductility	Softening Point
50/70	-	Unaged	1	3	2	-	1	-
		Aged	5	5	4	3	5	3
Bio-1	Waste edible oil	Unaged	7	6	7	-	7	-
		Aged	7	7	3	4	7	5
Bio-2	Tung oil	Unaged	5	5	6		5	-
		Aged	3	4	5	6	3	6
Bio-3	Biodiesel	Unaged	3	4	5	-	4	-
		Aged	2	2	8	7	2	8
Bio-4	Fish oil residue	Unaged	8	8	1	-	8	-
		Aged	6	8	1	2	6	2
Pio-L	Light fraction oil	Unaged	6	7	8	-	6	-
		Aged	8	6	6	1	8	1
Pio-M	Middle fraction oil	Unaged	2	1	4		2	-
		Aged	1	1	7	8	1	7
Pio-H	Heavy fraction oil	Unaged	4	2	3	-	3	-
		Aged	4	3	2	5	4	4

Note: Only the aged asphalt samples have been ranked for penetration and softening point (according to their values from smallest to largest) because these two indicators have no significant difference in all the samples before aging. The indicators (*T_CR_*, *N_f_*, *J*_*nr*3.2_, and ductility) are all ordered from best to worst, whereas the penetration and softening points follow the order of their values (from smallest to largest).

**Table 5:** Correlation of the mechanical and fraction indicators.

Mechanical Indicators	Fractions Indicators					
	*σ*	Asphaltene Ratio	Resins Ratio	Aromatic Ratio	Saturated Ratio	Heavy/ Light Fraction
*T_CR_*	0.982 ***	0.956 ***	0.714 **	0.926 ***	0.276	0.821 **
*N_f_*	0.764 **	0.853 **	0.482 *	0.728 **	0.143	0.612 **
*J*_*nr*3.2_	0.857 **	0.742 **	0.891 **	0.827 **	0.609 **	0.925 ***
Penetration	0.905 ***	0.773 **	0.889 **	0.871 **	0.468 *	0.923 ***
Softening Point	0.778 **	0.694 **	0.789 **	0.777 **	0.495 *	0.831 **
Ductility	0.965 ***	0.959 ***	0.775 **	0.926 ***	0.367	0.859 **

Note: *R*^2^ > 0.9; strong correlation, marked ***. *R*^2^ in 0.6~0.9; correlation, marked **. *R*^2^ in 0.4~0.6; weak correlation, marked *. *R*^2^ < 0.4; weak correlation, not marked.

## Data Availability

Not applicable.
